# COVID-19 Vaccines and Public Anxiety: Antibody Tests May Be Widely Accepted

**DOI:** 10.3389/fpubh.2022.819062

**Published:** 2022-05-06

**Authors:** Leyuan Liu, Xiaoxiao Wang, Xiaoguang Li, Nan Li

**Affiliations:** ^1^Department of Infectious Diseases, Peking University Third Hospital, Beijing, China; ^2^Research Center of Clinical Epidemiology, Peking University Third Hospital, Beijing, China

**Keywords:** COVID-19, SARS-CoV-2, vaccine, antibody tests, public anxiety

## Abstract

**Background:**

More than 200 countries are experiencing the coronavirus disease (COVID-19) pandemic. COVID-19 vaccination strategies have been implemented worldwide, and repeat COVID-19 outbreaks have been seen. The purpose of this study was to investigate the impact of COVID-19 vaccination on the reduction of perceived anxiety and the association between public anxiety and antibody testing intention during the COVID-19 pandemic.

**Methods:**

Chinese adults aged 18 and over were surveyed using an anonymous online questionnaire in April and May 2021. The questionnaire collected sociodemographic characteristics, vaccination characteristics, perceived anxiety due to COVID-19, and attitudes toward future antibody testing after COVID-19 vaccination. Perceived anxiety was assessed on a visual analog scale (VAS). Multivariate logistic regression analysis was used to determine the factors influencing future antibody detection.

**Results:**

A total of 3,233 people were investigated, 3,209 valid questionnaires were collected, and the response rate was 99.3%. Of the 3,209 respondents, 2,047 were vaccinated, and 1,162 were unvaccinated. There was a significant difference in anxiety levels between vaccinated and unvaccinated respondents (24.9±25.4 vs. 50.0±33.1, respectively). With the local spread of COVID-19 in mainland China, the public anxiety VAS scores increased by 15.4±25.6 (SMD=120%) and 33.8±31.7 (SMD=49%) among vaccinated and unvaccinated respondents, respectively. Of the 2,047 respondents who were vaccinated, 1,626 (79.4%) thought they would accept antibody testing. Those who displayed more anxiety about acquiring COVID-19 disease were more likely to accept COVID-19 antibody testing. If the antibody test results showed protective antibodies, 1,190 (58.1%) were more likely to arrange travel plans in China, while 526 (25.7%) thought they would feel safer traveling abroad.

**Conclusion:**

COVID-19 vaccination strategies help reduce public anxiety. However, public anxiety may be elevated as the local transmission of COVID-19 occurs in mainland China, which is usually caused now by imported cases. Those who display more anxiety choose to have antibody testing. Improving the accessibility of COVID-19 antibody tests can help ease public anxiety and enhance the confidence of some people to participate in social activities.

## Introduction

To date, the global COVID-19 pandemic caused by SARS-CoV-2 has not been fully controlled. Globally, as of 7:07 pm CEST on 9 August 2021, there have been 202,608,306 confirmed cases of COVID-19, including 4,293,591 deaths, reported to the WHO^1^. The numbers of confirmed COVID-19 cases and deaths continue to climb. The epidemic has not only seriously threatened human safety, but it has also affected global economic development to a certain extent (1–4). In addition, COVID-19 has caused psychological stress, which can even lead to psychological crises. Therefore, there is a need to not only take precautions to avoid COVID-19 infections but also to take necessary measures in preserving mental health. The COVID-19 vaccine is an important and effective means to prevent and control the continuous outbreak of the epidemic (5–8). The world has achieved great success in vaccine development, and COVID-19 vaccines are relatively safe (9–11). The number of countries vaccinated against SARS-CoV-2 is climbing. According to reports, as of 7 January 2022, Chinese citizens had received a total of 2,887.772 million doses of COVID-19 vaccine, with a vaccine coverage rate of 89.54%. A total of 1,215.878 million people, or 86.25% of the total population, were vaccinated. However, there have been no studies examining the impact of COVID-19 vaccination status on perceived anxiety reduction or public concerns after vaccination. Research is urgently needed to address the above issues given the importance of COVID-19 vaccination in directing future evidence and public health policy.

In line with Rogers' (12) protection motivation theory (PMT), individuals in the presence of a health risk are more involved in healthy behaviors (12). Anxiety about the epidemic and the worry of being infected may be the driving force behind determining if the vaccine is protective. The COVID-19 antibody test provides a numerical value that indicates whether people may have antibodies to COVID-19. It is important to identify the relationship between anxiety and COVID-19 antibody testing intention. Further understanding the causes of their anxiety could help develop targeted persuasion in preserving mental health during the COVID-19 pandemic.

This study is a cross-sectional survey conducted in China using social media to describe perceived anxiety levels in vaccinated and nonvaccinated respondents to determine the impact of COVID-19 vaccination on anxiety reduction. In addition, the study will explore the association between COVID-19 anxiety and antibody testing intention and the impact of antibody testing on reducing public anxiety.

## Materials and Methods

### Study Design

A cross-sectional survey was conducted between April and May 2021. An anonymous online questionnaire was disseminated via WeChat, a Chinese multipurpose messaging, social media and mobile payment app with 1.225 billion users in China and developed by Tencent. Considering that the respondents from WeChat tend to be young and have a higher education level, as a supplement, we used the recruitment service provided by the Tencent questionnaire to recruit subjects aged ≥50 and with a lower education level (middle school and below). The Tencent questionnaire sample population covers over 1 million respondents whose personal information was confirmed, allowing for an authentic, diverse and representative sample.

Chinese respondents aged 18 years and above residing in China were eligible to participate in the survey. In general, 2,588 respondents were recruited via WeChat, 645 respondents were recruited via the Tencent questionnaire, and the final sample consisted of 3,209 respondents after quality control and manual check procedures to exclude incomplete and invalid questionnaires. WeChat and Tencent platforms do not charge any fees for the questionnaire release and respondents. In WeChat or Tencent questionnaire, each account can only be submitted once, which can avoid fraud or multiple times completed by one person. Prior to the interview, all respondents had signed informed consent. The study was approved by the Peking University Third Hospital Medical Science Research Ethics Committee (No. 2021-184-01).

### Measures

Based on the main purpose of the study, the researchers designed the self-report questionnaire. The main sections of the questionnaire are as follows: (1) sociodemographic characteristics, such as age, sex, employment status, education, personal income, and residence; (2) vaccination characteristics, such as flu vaccination history, attitudes toward herd immunity, vaccine type and producer; (3) perceived anxiety due to the perceived threat related to COVID-19 in different scenarios (current: China continues enforcing certain COVID-19-related restrictions, and the COVID-19 epidemic in China has been effectively controlled; hypothetical scenarios: As the local transmission of COVID-19 occurs in mainland China, which is usually now caused by imported cases); and perceived anxiety was assessed on a visual analog scale (VAS), with anchor words describing the extremes of anxiety from “not at all” to “extremely”. Respondents are asked to place a mark along the visual line to indicate the intensity of their anxiety. (4) Concerns about COVID-19, including fear of infection for individuals and family members, fear of COVID-19 restrictions being eased or tightened, fear that income may be affected, and fear that SARS-CoV-2 may mutate, the vaccine may be ineffective, and the vaccine may cause side effects; (5) acceptance, attitude, and preferences regarding future antibody testing after COVID-19 vaccination. All questions were closed-ended, with tick boxes provided for responses. Prior to its administration in the present study, the questionnaire was tested in a pilot study among people with or without a medical profession (data not published or included in this paper). Respondents were asked to qualitatively evaluate the intelligibility of the questions. The questionnaire was finalized until there were no new amendment suggestions. The reliability index was assessed for the “perceived anxiety levels” items using Cronbach's alpha (internal consistency coefficient). The alpha values were 0.87 and 0.76 for the nonvaccinated and vaccinated respondents, respectively, showing a satisfactory level of reliability. Factor analysis was used to evaluate the structural validity of “public concerns” items. Three components were extracted: fear of infection for individuals and family members, fear of SARS-CoV-2 mutation and an ineffective vaccine, worry about COVID-19 restrictions and impacts on income. These results showed that the questionnaire had a satisfactory level of validity.

### Sample Size

Our preliminary investigation indicated that 60% of the respondents were vaccinated and 80% of the vaccinated respondents would accept antibody testing. Therefore, 48% of the survey population would accept antibody testing. We calculated that a sample of 2,398 respondents would generate a 95% confidence interval estimate (CI), which is a range of likely values for the population proportion with precision (allowable error) of ±2% based on an estimated sample proportion of 48%. Given an anticipated dropout rate of 20%, the minimum sample size required is 2,998.

### Statistical Analysis

Descriptive statistics were performed to describe the sociodemographic characteristics, vaccination characteristics, perceived anxiety, and acceptance of future antibody testing. Independent *t* tests and chi-squared tests were applied to compare continuous and categorical baseline characteristics in the two groups (vaccinated vs. unvaccinated). Independent *t* tests were used to compare perceived anxiety VAS scores between the vaccinated and unvaccinated groups. The standardized mean difference (SMD), which expressed the effect size, was calculated. Antibody testing acceptance rates were compared among different groups according to sociodemographic and vaccination characteristics. Rate differences and 95% CIs were calculated to measure the magnitude of the effect. Univariate and multivariate logistic regression analyses were performed to identify factors influencing acceptance of future antibody testing. Odds ratios and 95% CIs were calculated. All data were analyzed using R, version 4.0.3. A *p* < 0.05 was considered statistically significant.

## Results

### Study Sample Characteristics

In total, 3,209 respondents completed the questionnaires, giving a response rate of 99.3%. A total of 3,176 respondents were located across 31 provinces or administrative regions of mainland China (Supplementary Table S1). Table 1 shows the basic characteristics of respondents by COVID-19 vaccination status (vaccinated: 2,047; unvaccinated: 1,162). The participants were aged 18 to 80 years, with an average age of 38.7 years old (SD: 12.5). COVID-19 vaccination coverage was highest among adults aged 35–44 years (71.9%) and lowest coverage was among adults aged 18–24 years (49.7%) and ≥65 years (56.0%). 63.5% of males and 63.9% of females were vaccinated. The reported proportion of COVID-19 vaccination was highest among health care personnel (72.4%) and lowest among students (46.0%). Respondents who had a master's degree or above showed the highest vaccination rates (74.1%), and vaccination rates increased with a higher income level (51.2, 62.0, 66.5, 70.9%). Respondents who lived in urban areas were more likely to accept the COVID-19 vaccines that respondents who lived in rural areas (65.1 vs. 55.9%, respectively).

**Table 1:** The basic characteristics of the 3,209 respondents in the survey.

**Characteristics**	**Unvaccinated**	**Vaccinated**	**t/χ^2^**	**P**
	**(*n =* 1,162)**	**(*n =* 2,047)**		
Age	37.9 ± 13.6	39.1 ± 11.8	−2.685	0.007
Age group			60.22	<0.001
18–24	200 (50.3)	198 (49.7)		
25–34	360 (36.3)	632 (63.7)		
35–44	193 (28.1)	494 (71.9)		
45–54	246 (33.0)	499 (67.0)		
55–64	100 (38.6)	159 (61.4)		
65 and above	44 (44.0)	56 (56.0)		
Gender			0.057	0.812
Male	446 (36.5)	777 (63.5)		
Female	716 (36.1)	1,270 (63.9)		
Employment status			94.199	<0.001
Employed (healthcare)	258 (27.6)	676 (72.4)		
Employed (non-healthcare)	583 (35.4)	1,066 (64.6)		
Students	184 (54.0)	157 (46.0)		
Retired	137 (48.1)	148 (51.9)		
Healthcare staff			42.062	<0.001
No	904 (39.7)	1,371 (60.3)		
Yes	258 (27.6)	676 (72.4)		
Education			82.584	<0.001
Middle school and below	153 (38.9)	240 (61.1)		
High school	282 (49.0)	294 (51.0)		
Associate or bachelor	498 (36.7)	858 (63.3)		
Master and above	229 (25.9)	655 (74.1)		
Income (CNY per month)			66.314	<0.001
0–2,000	293 (48.8)	307 (51.2)		
2,000–5,000	319 (38.0)	521 (62.0)		
5,000–10,000	265 (33.5)	525 (66.5)		
10,000 and above	285 (29.1)	694 (70.9)		
Residence			14.402	<0.001
Urban	954 (34.9)	1,783 (65.1)		
Rural	201 (44.1)	255 (55.9)		

*CNY, China Yuan*.

Table 2 shows the vaccination characteristics of the respondents. The reported proportion of flu vaccination history was found to be significantly lower in the nonvaccinated respondents (19.4%) than in the vaccinated respondents (42.5%). A total of 88.2% of the vaccinated respondents and 80.9% of the nonvaccinated respondents thought that herd immunity would be an effective way to prevent and control COVID-19.

**Table 2:** The vaccination characteristics of the 3,209 respondents in the survey.

**Characteristics**	**Unvaccinated**	**Vaccinated**	**χ^2^**	***P***
	**(*n =* 1,162)**	**(*n =* 2,047)**		
Flu vaccination history			175.867	<0.001
No/Unsure	937 (80.6)	1,178 (57.5)		
Yes	225 (19.4)	869 (42.5)		
Attitudes toward herd immunity			31.786	<0.001
Noneffective/unsure	222 (19.1)	242 (11.8)		
Effective	940 (80.9)	1,805 (88.2)		
Vaccine type				
Unknown	—	597 (29.2)	—	—
Inactivated virus vaccine	—	1,346 (65.8)	—	—
Adenovirus vector vaccine	—	31 (1.5)	—	—
Recombinant subunit vaccine	—	32 (1.6)	—	—
mRNA vaccine	—	24 (1.2)	—	—
Others	—	17 (0.8)	—	—
Vaccine Producer				
Unknown	—	607 (29.7)	—	—
Sinopharm Group^a^	—	430 (21.0)	—	—
Beijing Sinovac^b^	—	844 (41.2)	—	—
CanSino^c^	—	20 (1.0)	—	—
Zhifei Longcom^d^	—	51 (2.5)	—	—
Others	—	95 (4.6)	—	—

a *Inactivated SARS-CoV-2 vaccine (Vero cells) from Beijing Institute of Biological Products/Sinopharm (abbreviation BBIBP-CorV);*

b *Inactivated SARS-CoV-2 vaccine (Vero cells) from Beijing Sinovac Biotech Co Ltd. (abbreviation Sinovac-CoronaVac);*

c *The recombinant adenovirus type 5 vector vaccine from CanSino Biological Inc./Beijing Institute of Biotechnology;*

4 *The recombinant protein vaccine (CHO cells) from Anhui Zhifei Longcom Biopharmaceutical/Institute of Microbiology, Chinese Academy of Sciences*.

### Impact of COVID-19 Vaccination Status on Perceived Anxiety

**Figure 1:**
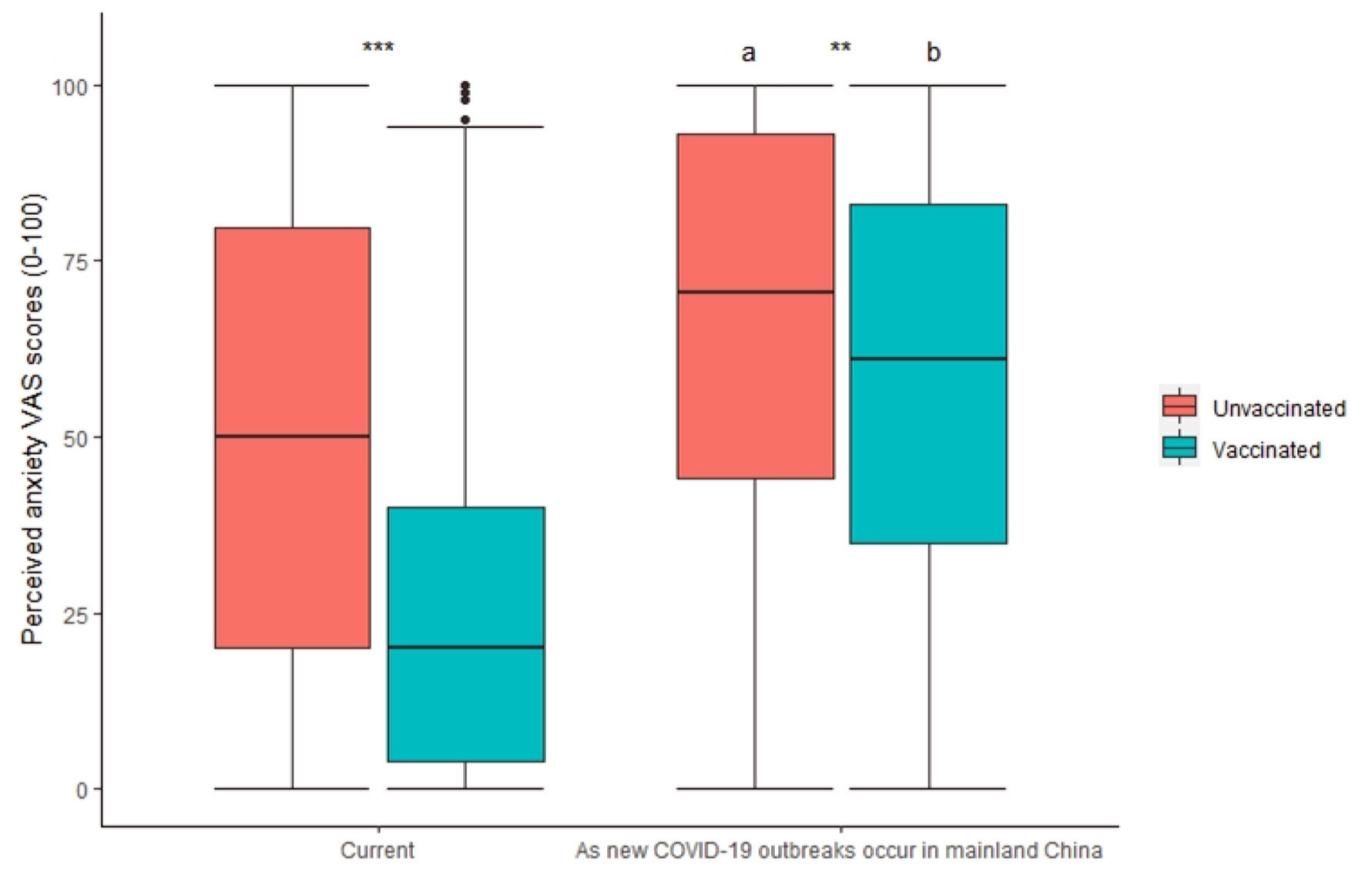
Impact of COVID-19 vaccination status on perceived anxiety VAS scores. a, b, *p* < 0.05 in comparison with “Current”; ^**^p < 0.01; ^***^p < 0.001.

In terms of perceived anxiety due to the perceived threat related to COVID-19, the anxiety levels of the vaccinated respondents were found to be significantly lower than those of the nonvaccinated respondents (24.9 ± 25.4 vs. 50.0 ± 33.1, respectively; Figure 1). The results remained robust after adjusting for a wide range of confounders (Supplementary Table S2). The difference in anxiety VAS scores between the vaccinated and nonvaccinated respondents was 25.1 ± 1.1 (SMD=85%). Public anxiety may be elevated as the local transmission of COVID-19 occurs in mainland China, which is usually now caused by imported cases, irrespective of whether the respondents have been vaccinated, because the respondents worry about the risk of being infected (Figure 1). Public anxiety VAS scores increased by 15.4 ± 25.6 (SMD=120%) and 33.8 ± 31.7 (SMD = 49%) in the vaccinated and nonvaccinated respondents, respectively.

**Figure 2:**
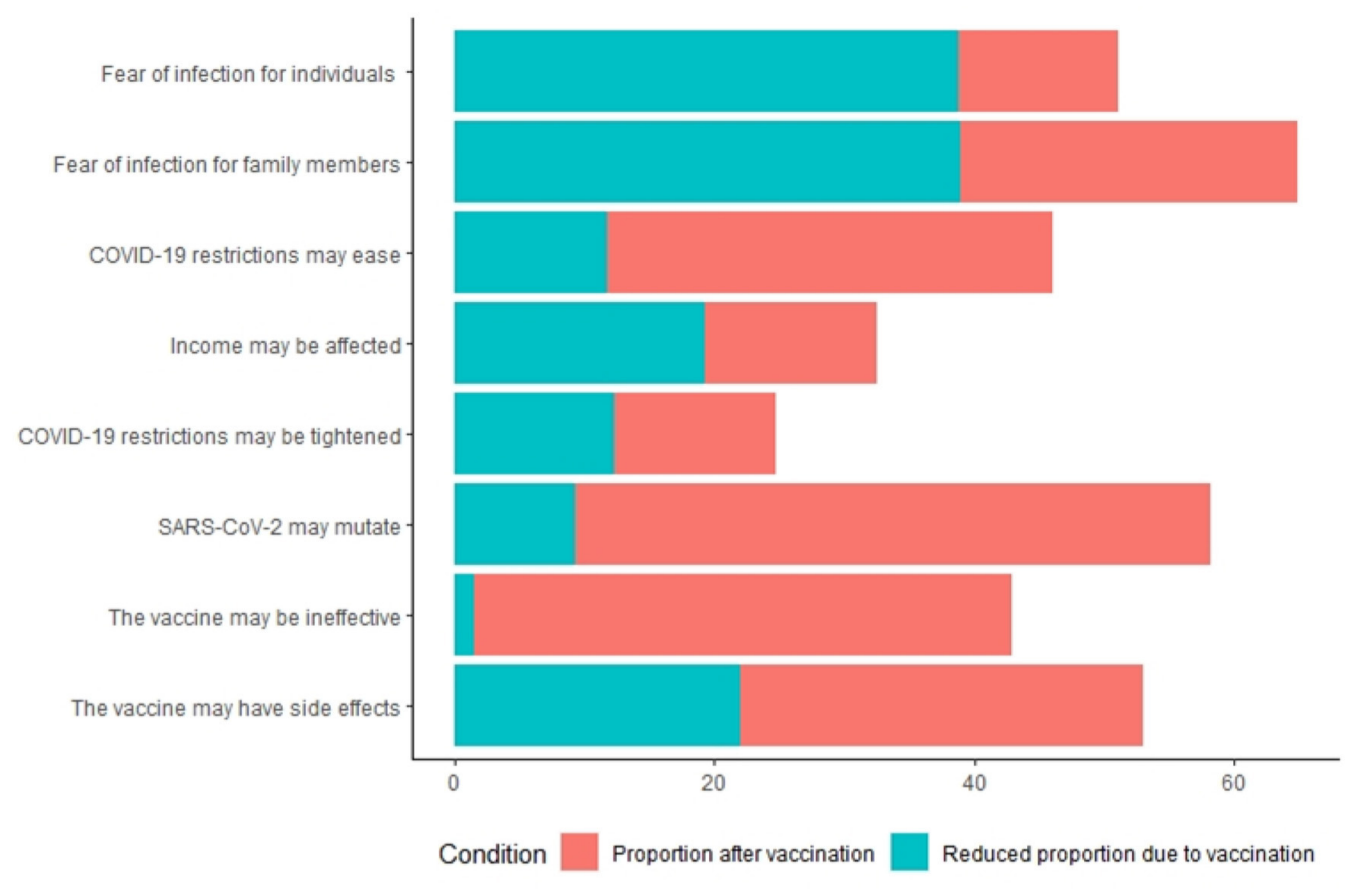
Impact of COVID-19 vaccination status on public concerns.

Figure 2 shows the concerns about the COVID-19 of the respondents. In terms of the causes of perceived anxiety about COVID-19, 51 and 65% of nonvaccinated respondents reported fear due to the perceived risk to personal health and of infection of family members, respectively; 58 and 53% reported fear of SARS-CoV-2 mutations and adverse reactions to vaccines, respectively. Of the vaccinated respondents, 49% reported fear of SARS-CoV-2 mutations.

In most cases, nonvaccinated respondents reported greater concern about COVID-19 than vaccinated respondents (Supplementary Table S3). There is one exception: concerns about the vaccination's effectiveness did not ease after the vaccination was administered (Supplementary Table S3). Forty-three percent and 41% of the nonvaccinated and vaccinated respondents, respectively, were concerned that obtaining the COVID-19 vaccine is not effective against SARS-CoV-2.

### Acceptance, Preferences and Impact Factors of Future Antibody Testing After COVID-19 Vaccination

Of the 2,047 respondents who were vaccinated, 1,626 (79.4%) thought they would accept antibody testing after COVID-19 vaccination. Health care respondents, respondents who thought that herd immunity would be effective against SARS-CoV-2 and those who displayed more anxiety about acquiring COVID-19 disease were more likely to accept COVID-19 antibody testing (Table 3). In addition, if the antibody test results showed protective antibodies, 1,190 (58.1%) were more likely to arrange travel plans in China, while 526 (25.7%) thought they would feel safer traveling abroad.

**Table 3:** Influencing factors on antibody testing after COVID-19 vaccination.

**Characteristics**	**Antibody testing acceptance**	**Rate difference (95% CI)**	**Crude odds ratio (95% CI)**	**Adjusted odds ratio (95% CI)**
Age group				
18–24	151 (76.3)	4.8% (3.9%, 5.7%)	1.285 (0.660,2.501)	1.331 (0.647,2.737)
25–34	511 (80.9)	9.4% (8.7%, 10.2%)	1.689 (0.915,3.117)	1.690 (0.888,3.216)
35–44	393 (79.6)	8.1% (7.3%, 8.9%)	1.556 (0.838,2.892)	1.545 (0.810,2.950)
45–54	397 (79.6)	8.1% (7.3%, 8.9%)	1.557 (0.838,2.892)	1.534 (0.812,2.899)
55–64	127 (79.9)	8.5% (7.5%, 9.4%)	1.587 (0.790,3.189)	1.562 (0.761,3.206)
≥65	40 (71.4)	Reference	Reference	Reference
Gender				
Male	628 (80.8)	2.2% (2.2%, 2.3%)	1.149 (0.919,1.436)	1.153 (0.913,1.456)
Female	998 (78.6)	Reference	Reference	Reference
Occupation				
Healthcare	560 (82.8)	5.1% (5.0%, 5.1%)	**1.381 (1.090,1.751)**	**1.538 (1.170,2.022)**
Non-healthcare	1,066 (77.8)	Reference	Reference	Reference
Education				
Middle school and below	192 (80.0)	1.7% (1.5%, 1.9%)	1.107 (0.767,1.598)	1.340 (0.799,2.247)
High school	232 (78.9)	0.6% (0.4%, 0.7%)	1.036 (0.740,1.450)	1.344 (0.865,2.087)
Associate or bachelor	689 (80.3)	2.0% (1.9%, 2.1%)	1.129 (0.878,1.450)	1.291 (0.983,1.696)
Master and above	513 (78.3)	Reference	Reference	Reference
Income (CNY per month)				
0–2,000	239 (77.9)	−3.1% (−3.3%, −3.0%)	0.826 (0.594,1.148)	0.881 (0.557,1.392)
2,000–5,000	405 (77.7)	−3.2% (−3.4%, −3.1%)	0.820 (0.620,1.085)	0.823 (0.580,1.166)
5,000–10,000	420 (80.0)	−1.0% (−1.1%, −0.9%)	0.940 (0.706, 1.250)	0.918 (0.677, 1.245)
≥10,000	562 (81.0)	Reference	Reference	Reference
Residence				
Rural	210 (82.4)	3.4% (3.2%, 3.5%)	1.243 (0.883, 1.749)	1.351 (0.892, 2.044)
Urban	1,408 (79.0)	Reference	Reference	Reference
Flu vaccination history				
Yes	708 (81.5)	3.5% (3.5%, 3.6%)	1.245 (1.000,1.551)	1.238 (0.988,1.552)
No/Unsure	918 (77.9)	Reference		
Attitudes toward herd immunity				
Effective	1,456 (80.7)	10.4% (10.2%, 10.6%)	**1.767 (1.310, 2.383)**	**1.872 (1.372, 2.553)**
Noneffective/Unsure	170 (70.2)	Reference	Reference	Reference
Perceived more anxiety from COVID-19				
Yes	1,240 (80.6)	4.6% (4.5%, 4.7%)	**1.311 (1.032, 1.666)**	**1.365 (1.067, 1.747)**
No	386 (76.0)	Reference	Reference	Reference

*CI, confidence interval; CNY, China Yuan. The bold values are statistically significant (P < 0.05)*.

In terms of concerns about antibody testing after COVID-19 vaccination, 916 (44.7%) were concerned about the cost of antibody testing. Free COVID-19 antibody testing would cause 778 (84.9%) respondents to accept antibody tests. A total of 1,016 (49.6%) were concerned about the diagnostic accuracy of antibody tests for COVID-19. For 850 (83.7%) respondents, they would accept antibody testing after COVID-19 vaccination if they knew that antibody testing is reliable. In addition, 673 (32.9%) of respondents were concerned about convenient access to antibody tests, but 579 (86.0%) reported that if easy access to antibody testing were guaranteed, they would accept antibody testing.

## Discussion

It is not easy to control the rapid development of COVID-19, which has heavily impacted the travel, life and income of populations worldwide (13). From the perspective of epidemiology, it is very important to control the source of infection, cut off the transmission route and protect the susceptible population in the prevention and control of the epidemic (14–17). In addition to COVID-19 vaccination, it is necessary to maintain self-awareness, such as wearing a mask, washing hands frequently, disinfection, and maintaining social distancing (14, 18, 19). At present, many countries have strictly implemented the abovementioned measures, but the situation of epidemic control still fluctuates. The COVID-19 vaccine, which the WHO has recognized as safe and effective, is critical to ending the outbreak (20–22). The results also showed that respondents who had been vaccinated had significantly lower levels of anxiety than those who had not. This is another example of the importance and necessity of COVID-19 vaccination.

At present, despite the constant adjustment of epidemic prevention and control policies in various countries around the world, COVID-19 still has a trend of repeated outbreaks. As COVID-19 cases emerge and restrictions are relaxed, more respondents will experience increased anxiety and opt for vaccination. Before the launch of the vaccine, China's willingness to vaccinate against COVID-19 was as high as 90% (23, 24). In this survey, the COVID-19 vaccine coverage rate of citizens was 63.79%. Demographic and sociological characteristics affected the vaccination rate, and the degree of vaccine recognition also obviously affected the vaccination rate of the respondents. Higher levels of education and income were associated with higher rates of COVID-19 vaccination, with the highest coverage among medical personnel. In China, COVID-19 vaccination is free of charge, and the reason why income influences the COVID-19 vaccination rate is related to education level. People with higher education levels generally have higher incomes and higher awareness of the COVID-19 vaccine. Therefore, it is very important to understand the COVID-19 vaccine correctly. In view of this, governments should make maximum efforts to promote the progress of vaccination by disseminating vaccine knowledge and the advantages and disadvantages of vaccination through multiple channels. Advancing closer to herd immunity and reducing the population's anxiety can promote the steady development of the economy. At the early stage of mass vaccination, 55.3% of people reported wanting to be vaccinated immediately (25). But as of October 25, 2021, 2.249 billion doses of the vaccine have been administered in China, which means that the vaccination rate has reached 75.2% (26). The survey shows that public concern about the risk of infection may increase during repeated outbreaks of COVID-19 and thus increase the vaccination rate. This may explain why actual vaccination rates are higher than early-stage immediate vaccination willingness.

During the COVID-19 pandemic, many people became infected with the virus. Concerns have been raised as to whether infected individuals gained protective immunity against SARS-CoV-2. Multiple studies have confirmed increased COVID-19 antibody to SARS-CoV-2 in infected persons, especially after exposure to COVID-19 patients (27, 28). The antibody is glycosyl-based globulins that are synthesized and secreted by stimulating the differentiation and proliferation of B cells into plasma cells after antigen entering the body. That can combine with antigens on the surface of pathogenic microorganisms to prevent them from adhering to target cell receptors and invading cells. Existing vaccines against hepatitis B, hepatitis A, measles and polio all produce antibodies to protect against the virus (29–32). A recent case study documented that SARS-CoV-2 reinfection was associated with weakened COVID-19 antibody (33). Data from a Norwegian survey of health care workers infected with novel coronavirus infections showed a higher proportion of SARS-CoV-2 seropositivity than RT–PCR positivity (34). This undoubtedly highlights the importance of antibody detection. Testing is critical for diagnosing prior infections and predicting future immunity brought by SARS-CoV-2 antibodies.

The epidemic has not only affected citizens' lives, travel and work, but it has also increased their mental and psychological stress (35–37). The level of public anxiety is far higher than at any other time. In the current study, anxiety levels were significantly lower among vaccinated respondents than among nonvaccinated respondents. Whether protective antibodies can be produced after vaccination against COVID-19 is an important issue of public concern (38, 39). In the study, 1,626 people (79.4%) who had been vaccinated against COVID-19 thought they would be tested for antibodies. A high willingness to test for COVID-19 antibody suggests that vaccine protection at the individual level can help further alleviate public anxiety. The results indicated that when antibody test results showed protective antibodies, 58.1% of people would travel around China, and 25.7% of people would travel abroad. Therefore, improving the accessibility of COVID-19 antibody tests can help ease public anxiety and enhance the confidence of some people to participate in social activities.

There are many factors influencing antibody testing, including the cost, accuracy and convenience of antibody testing. The accuracy of antibody detection, that is, the validity and reliability of antibody detection, is particularly important. After screening samples using the Diazyme SARS-CoV-2 IgG serological assay, positive samples were reanalyzed using the neutralization assay, the Roche total immunoglobin assay, and the Abbott IgG assay A positive correlation was observed between the size of SARS-CoV-2 serological test results and neutralization activity in COVID-19 patients. COVID-19 antibody is considered protective (40). COVID-19 antibody levels against SARS-CoV-2 can be used to assess acquired protective immunity in COVID-19 patients or vaccinators (41). A study used the plaque reduction neutralization test as a reference to evaluate the diagnostic performance of six commercial serological tests used to monitor SARS-CoV-2 antibodies. The results showed support for VIDAS SARS-CoV-2 IgG, Euroimmun anti-SARS-CoV-2 ELISA IgG, and Euroimmun anti-SARS-CoV-2 QuantiVac ELISA IgG, and Microblot-array COVID-19 IgG assay was performed to monitor COVID-19 antibody responses following natural SARS-CoV-2 infection (42). Studies are also underway on antibody testing after vaccination (43–45). Antibody testing can identify those who have been immunized, providing a basis for the follow-up implementation of refined epidemic prevention and control measures, reducing public anxiety, easing travel restrictions, and preparing for a full exit from the struggle to contain the epidemic and prevent future outbreaks.

The biggest concern is that it is difficult to test for COVID-19 antibodies. In addition to the above reasons, the public's understanding of COVID-19 antibody testing is still insufficient. The SARS-CoV-2 antibody test has been recognized as a widely used tool in the surveillance and control of the COVID-19 pandemic to better target populations at risk of exposure to the disease (46, 47). Currently, novel coronavirus IgG and IgM test kits are being used in clinical medicine. Some kits are available to detect total antibodies, including IgA in addition to IgG and IgM (48–50). It has been shown that patients who test positive for COVID-19 antibodies have lower in-hospital mortality than those who test negative (51). Some scholars suggest that in-depth studies should be conducted on people with disabilities and special groups to address the problem of vaccine hesitating (52, 53). The importance of early and proactive COVID-19 vaccination education efforts for the public has also been raised (54, 55). For the promotion of COVID-19 antibody testing, scholars and experts should actively address public concerns about its effectiveness, convenience and cost. The government and relevant institutions can actively promote the feasibility of COVID-19 antibody testing, such as giving away brochures for COVID-19 antibody testing after vaccination, or making novel animations for propaganda and education on various social platforms. The promotion of COVID-19 vaccination and universal public acceptance of COVID-19 antibody testing complement each other and together build an important wall of protection against COVID-19.

This is the first study to examine the impact of COVID-19 antibody testing on vaccination intentions and on reducing public anxiety during the pandemic. At present, the novel coronavirus is mutating, resulting in multiple outbreaks, which also contribute to continuing anxiety. In view of the high number of infections of COVID-19 since its outbreak, we should accelerate the research and development and promotion of antibody testing to confirm the effectiveness of the COVID-19 vaccine, increase people's confidence in vaccination and reduce their anxiety about the epidemic. Regarding the survey method, network surveys have certain limitations. For example, people who do not use the internet cannot be covered, and investigators' suspicions cannot be answered in person. The age of the survey population was standardized according to China's national census standards. There was no significant difference in vaccine coverage before and after standardization (Table 4). In spite of this, subjects responding to the survey were not nationally representative and may not be generalizable to all Chinese adults in China. Due to the limited representativeness of the present study's sample, further investigation is needed in the future. This survey did not evaluate the information accessibility of each subject or the communication campaign, which may confound the effect of antibody detection on perceived anxiety reduction. In addition, there have been no clear reports confirming the relationship between antibody levels and protection provided by vaccination or recent recovery from infection. It is important to mention all these limitations. Therefore, further research and investigation are needed.

**Table 4:** The process of calculating age-standardized vaccination rate.

**Age (year)**	**Observed acceptance rates (%)**	**Population according to census 2016**	**Expected number**
18–24	49.7%	147625815	73370030
25–34	63.7%	215992522	137587237
35–44	71.9%	191968262	138025180
45–54	67.0%	235389183	157710753
55–64	61.4%	174783724	107317207
≥65	56.0%	190635280	106755757
Total	63.8%	1156394786	720766163

*The age- standardized vaccination rate equals 62.3% (720766163/1156394786)*.

## Data Availability Statement

The original contributions presented in the study are included in the article/Supplementary Material, further inquiries can be directed to the corresponding author/s.

## Ethics Statement

The study was approved by the Peking University Third Hospital Medical Science Research Ethics Committee (No. 2021-184-01).

## Author Contributions

All authors listed have made a substantial, direct, and intellectual contribution to the work and approved it for publication.

## Funding

This work was supported by the National Natural Science Foundation of China (Project No. 81701067) and National Major Science and Technology Projects (Project No. 2018ZX10732401-003).

## Conflict of Interest

The authors declare that the research was conducted in the absence of any commercial or financial relationships that could be construed as a potential conflict of interest.

## Publisher's Note

All claims expressed in this article are solely those of the authors and do not necessarily represent those of their affiliated organizations, or those of the publisher, the editors and the reviewers. Any product that may be evaluated in this article, or claim that may be made by its manufacturer, is not guaranteed or endorsed by the publisher.

## Footnotes

^1^ https://www.who.int/emergencies/diseases/novel-coronavirus-2019
