# Supplementary material for: COVID-19 Vaccines and Public Anxiety: Antibody Tests May Be Widely Accepted
Source: Front Public Health. 2022 May 6;10:819062. doi: 10.3389/fpubh.2022.819062 (PMC9120666; doi:10.3389/fpubh.2022.819062)
Supplement: Supplementary file 1 [file Data_Sheet_1.docx]

Table S1. The provincial distribution of respondents (n = 3209) of the survey

| Provincial distribution | Frequency | Percent |
| --- | --- | --- |
| Anhui | 30 | 0.9 |
| Beijing | 1256 | 39.1 |
| Fujian | 29 | 0.9 |
| Gansu | 14 | 0.4 |
| Guangdong | 147 | 4.6 |
| Guangxi | 9 | 0.3 |
| Guizhou | 15 | 0.5 |
| Foreign | 13 | 0.4 |
| Hainan | 41 | 1.3 |
| Hebei | 318 | 9.9 |
| Henan | 202 | 6.3 |
| Heilongjiang | 54 | 1.7 |
| Hubei | 57 | 1.8 |
| Hunan | 45 | 1.4 |
| Ji Lin | 34 | 1.1 |
| Jiangsu | 81 | 2.5 |
| Jiangxi | 37 | 1.2 |
| Liaoning | 68 | 2.1 |
| Inner Mongolia | 59 | 1.8 |
| Ningxia | 9 | 0.3 |
| Qinghai | 3 | 0.1 |
| Shandong | 101 | 3.1 |
| Shanxi | 70 | 2.2 |
| Shaanxi | 48 | 1.5 |
| Shanghai | 171 | 5.3 |
| Sichuan | 59 | 1.8 |
| Tianjin | 54 | 1.7 |
| Tibet | 30 | 0.9 |
| Hong Kong | 20 | 0.6 |
| Xinjiang | 10 | 0.3 |
| Yunnan | 21 | 0.7 |
| Zhejiang | 78 | 2.4 |
| Chongqing | 26 | 0.8 |
| Total | 3209 | 100 |

Table S2. Effect of COVID-19 vaccination status on perceived anxiety levels after adjusting for a wide range of confounders

|  | B | SE | t | P |
| --- | --- | --- | --- | --- |
| COVID-19 vaccination status | -24.760 | 1.094 | -22.640 | **<0.001** |
| Age group |  |  |  |  |
| 18-24 | -4.507 | 3.323 | -1.356 | 0.175 |
| 25-34 | 1.818 | 3.039 | 0.598 | 0.550 |
| 35-44 | 1.527 | 3.082 | 0.495 | 0.620 |
| 45-54 | 0.491 | 3.008 | 0.163 | 0.870 |
| 55-64 | 8.831 | 3.327 | 2.655 | 0.008 |
| ≥65 |  |  |  |  |
| Male | -2.293 | 1.048 | -2.188 | 0.029 |
| Healthcare staff | 0.608 | 1.254 | 0.485 | 0.628 |
| Education |  |  |  |  |
| Middle school and below | 9.320 | 2.339 | 3.985 | <0.001 |
| High school | 9.424 | 1.958 | 4.813 | <0.001 |
| Associate or bachelor | 4.574 | 1.284 | 3.562 | <0.001 |
| Master and above |  |  |  |  |
| Income (CNY per month) |  |  |  |  |
| 0-2000 | 2.788 | 2.102 | 1.326 | 0.185 |
| 2000-5000 | 2.619 | 1.614 | 1.623 | 0.105 |
| 5000-10000 | 2.899 | 1.402 | 2.068 | 0.039 |
| ≥10000 |  |  |  |  |
| Rural | 0.527 | 1.698 | 0.310 | 0.756 |
| Flu vaccination history | 2.691 | 1.083 | 2.485 | 0.013 |
| Attitudes towards herd immunity | -3.271 | 1.431 | -2.286 | 0.022 |

CI, confidence interval; CNY, China Yuan

Table S3. The concerns about the COVID-19 between the unvaccinated and vaccinate respondents

| Concerns | Unvaccinated  (n=1162) | Vaccinated  (n=2047) | χ^2^ | P |
| --- | --- | --- | --- | --- |
| Fear of infection for individuals | 594 (51.1) | 252 (12.3) | 575.047 | <0.001 |
| Fear of infection for family members | 755 (65) | 532 (26) | 468.981 | <0.001 |
| COVID-19 restrictions may ease | 536 (46.1) | 704 (34.4) | 43.056 | <0.001 |
| Income may be affected | 378 (32.5) | 272 (13.3) | 169.913 | <0.001 |
| COVID-19 restrictions may be tightened | 288 (24.8) | 255 (12.5) | 80.129 | <0.001 |
| COVID-19 may mutate | 678 (58.3) | 1004 (49) | 25.705 | <0.001 |
| The vaccine may be ineffective | 498 (42.9) | 847 (41.4) | 0.666 | 0.414 |
| The vaccine may have side effects | 616 (53) | 635 (31) | 150.701 | <0.001 |
